# Recurrence risk after Ivor Lewis oesophagectomy for cancer

**DOI:** 10.1186/1749-8090-8-215

**Published:** 2013-11-21

**Authors:** Mael Chalret du Rieu, Thomas Filleron, Benoit Beluchon, Marine Humeau, Charles-Henri Julio, Eric Bloom, Laurent Ghouti, Sylvain Kirzin, Guillaume Portier, Bernard Pradère, Nicolas Carrère

**Affiliations:** 1 Department of Digestive Surgery, Toulouse Purpan University Hospital, Toulouse III University, place du Dr Baylac, 31059 Toulouse France; 2 Department of Biostatistics, Institut Claudius Regaud, 20-24 rue du Pont Saint Pierre, 31052 Toulouse, France

**Keywords:** Oesophageal carcinoma, Oesophagectomy, Ivor-Lewis, Lymph node ratio, Tumor stenosis

## Abstract

**Objective:**

The aim of this study was to analyze the profile of tumor recurrence for patients operated on for cancer of oesophagogastric junction or oesophagus by Ivor-Lewis oesophagectomy.

**Methods:**

Patients undergoing potentially curative Ivor-Lewis oesophageal resection between January 1999 to December 2008 at a single center institution were retrospectively analyzed. Their clinical records, details of surgical procedure, postoperative course, pathological findings, recurrence and long term survival were reviewed retrospectively. Univariate and multivariate survival analyses were performed.

**Results:**

One hundred and twenty patients were analyzed. Fifty three patients (44%) presented recurrence during median follow-up of 58 months. Five-year relapse free survival (RFS) rate was 51% (95%CI = [46; 65%]). On multivariate analysis, pT stage > 2 (HR = 2.42, 95%CI = [1.22; 4.79] p = 0.011), positive lymph node status (HR = 3.69; 95% CI = [1.53; 8.96] p = 0.004) and lymph node ratio > 0.2 (HR = 2.57; 95%CI = [1.38; 4.76] p = 0.003) were associated with a poorer RFS and their combination was correlated to relapse risk. Moreover, preoperative tumor stenosis was associated with an increased risk of local recurrence (HR = 3.46; 95% CI = [1.38; 8.70] p = 0.008) whereas poor or undifferentiated tumor was associated with an increased risk of distant recurrence (HR = 3.32; 95% CI = [1.03; 10.04] p = 0.044).

**Conclusion:**

pT stage > 2, positive lymph node status and lymph node ratio > 0.2 are independent prognostic factors of recurrence after Ivor-Lewis surgery for cancer. Their combination is correlated with an increasing risk of recurrence that may argue favorably, in addition with preoperative tumor stenosis assessment, for adjuvant treatment or reinforced follow-up.

## Background

Oesophageal cancer is a major public health concern as it is the fourth cause of cancer death after lung, colorectal and prostate cancers. Without contraindication of resectability or operability, surgery is the standard treatment of curative intent. However, in spite of optimal R0 resection, overall 5-year survival is poor, about 20 to 30%, because of frequent tumor recurrence [1]. Thus, exclusive chemoradiotherapy has become an alternative to surgical treatment, with a comparable overall survival in locally advanced squamous cell carcinoma [2,3]. However, ratio between adenocarcinoma and squamous cell carcinoma incidence is changing with an increasing incidence of adenocarcinoma developed on Barrett oesophagus in Western countries [4]. Regarding adenocarcinoma, combination of neo-adjuvant treatment seems to provide improved survival despite of its own morbi-mortality [5]. Study of tumor relapse could therefore allow adaptation and targeting perioperative treatment to patients with high risk of recurrence. The aim of this study was to analyze the profile of tumor recurrence in a homogenous group of patients operated on for cancer of oesophagogastric junction or oesophagus by Ivor-Lewis oesophagectomy.

## Methods

From January 1999 to December 2008, 120 consecutive patients underwent an Ivor-Lewis oesophagectomy for cancer at a single institution. Their clinical records were reviewed retrospectively for age, sex, Body Mass Index (BMI), nutritional factors, American Society of Anesthesiologists (ASA) score, symptoms at diagnosis, preoperative treatment, details of the surgical procedure, pathological findings, postoperative course, recurrence and long term survival. The research was carried out in compliance with the Helsinki Declaration and approved by the Comité de Protection des Personnes (CPP) Sud Ouest (Toulouse, France).

### Preoperative evaluation

Operability and resectability criteria were those usually used in oncologic guidelines [6]. All surgical indications were validated in pluridisciplinary meeting. Endoscopy, computed tomography and barium swallows were systematically performed. Ultrasonographic endoscopy (EUS) was performed systematically as soon as it was technically available and when there was no non-traversable strictures. Positron emission tomography (PET) was done depending on the suspicion of metastatic extension. Features of lymph nodes on EUS and computed tomography, including size 1 cm or more, rounded shape, well demarcated borders, and heterogeneous patterns, were used to describe the nodes as benign or malignant.

Preoperative tumor stenosis was defined as a difficulty or inability for the fiberscope (11.6 mm standard diameter) to pass through the lesion and/or at least a hemi-circumferential narrowing light on barium swallow (both sides of the oesophagus narrowed by tumor noticed on at least one radiologic incidence).

Neo-adjuvant chemotherapy (CT) (Platin and 5 Fluorouracil) or chemoradiotherapy (CRT) (45 to 50 Gy and concomitant Platin/5 fluorouracil chemotherapy) was delivered in case of locally advanced cancer (T stage ≥ 3 and/or N stage ≥ 1). CRT was preferred for high volume tumors and/or for limited resection margins.

Clinical response was defined by regression of dysphagia and tumor size on post induction computed tomography. Endoscopic control, EUS nor PET were not routinely performed.

Immunonutrition was systematically delivered for seven preoperative days.

### Surgical procedure

Conventional orotracheal intubation was performed without selective bronchial intubation. Gastrolysis was done through laparoscopy or midline laparotomy. Thoracotomy was done through a right posterolateral thoracotomy in the fifth intercostal space. Gastric transplant was created in the chest and anastomosed mechanically to the oesophagus. An extended upper abdominal lymphadenectomy was routinely performed comprising en-bloc resection of the nodal tissue along the common hepatic and proximal splenic arteries together with that at the origins of the left gastric artery and celiac axis. The lesser omentum was divided, encompassing the nodes along the lesser curve and an en-bloc hiatal dissection was performed removing the left and right paracardial stations and the respective crura. Within the thorax, standard lymphadenectomy was routinely performed that comprised middle and lower paraoesophageal nodes, paratracheal, carinal, left and right bronchial nodes and left recurrent laryngeal nerve chain. No cervical lymphadenectomy or three-field lymphadenectomy was undertaken even for tumors of middle third oesophagus. Patients were extubated in recovery room and then transferred to the ward.

### Postoperative data

All tumors were staged post surgically by the TNM classification system of the American Joint Committee on Cancer 2002 for oesophageal or gastric cancer depending on tumor localization. The resection was designated R0 when it was thought that both macroscopic and microscopic clearance had been achieved; R1 when it was microscopically incomplete (margin inferior to one millimeter), and there was histologic evidence of invasion of the longitudinal or lateral margins; and R2 when it was macroscopically incomplete, with macroscopic residual tumor after surgery. Median lymph node ratio was defined by the ratio between number of positive lymph nodes and number of resected lymph nodes.

Operative mortality and morbidity was defined as any death or complication during the first 30 days after operation. A major complication was defined as any complication > 3 on Clavien Dindo classification.

Follow up data were obtained from patient charts, tumor registry and referring physicians. A clinical examination was carried out every three to six months for three years. Thoraco-abdominal computed tomography was performed at least twice a year for 3 years or according to symptoms. Endoscopy was done in case of dysphagia.

For recurrence study, final date of follow-up was scheduled for March 31, 2010. Histologic, cytologic, or unequivocal radiologic proof was required before recurrence was diagnosed. Recurrence was classified into three categories: local recurrence (occurring at the surgical resection site or on cervical site), distant recurrence (in case of distant metastasis) and disseminated (in case of local and distant recurrence). Operative deaths and deaths that were not related to cancer were included in the survival statistics.

### Statistical analysis

Data were summarized by frequency and percentage for categorical variables and by median and range for continuous variables. All survival times were calculated from the date of surgery. Relapse free survival (RFS) and overall survival (OS) were estimated by the Kaplan-Meier methods using the following first-event definitions: loco-regional or distant recurrence for relapse free survival and death from any cause for overall survival. Univariate analysis was performed using the Log-Rank test. The following three step algorithm using Cox proportional hazard modeling was performed:

Step 1: Influence of pre-operative variables with a p-value <0.05 in univariate analysis was evaluated.

Step 2: Influence of pathological variables with a p-value <0.05 in univariate analysis was evaluated.

Step 3: Influence of pre-operative and pathological variables statistically significative in step 1 and 2 was evaluated.

Competing risks multivariate analysis was conducted using the Fine & Gray model in order to evaluate the influence of different variables on the cumulative incidence in the presence of competing risks (i.e local recurrence vs distant relapse).

All p-values reported were two-sided. For all statistical tests, differences were considered significant at the 5% level. Statistical analyses were performed using the STATA 10.0 software.

## Results

### Preoperative results

One hundred and twenty patients were included. Median age at surgery was 62 years [27-79 years] (Table 1). Sex ratio male: female was 4: 1. Dysphagia was the main symptom (68%) and 16% of patients were asymptomatic among them 11.6% were diagnosed for Barrett oesophagus follow-up and 5% (n = 6) on an examination prescribed for other indication. Severe weight loss (weight loss exceeding 10% of usual weight in one month or 15% in six months) was present in 20% of patients as 32% were overweight (BMI > 25 Kg/m^2^) and 6% were obese (BMI > 30 Kg/m^2^). Median BMI was 23.5 Kg/m^2^ [12.5-43.3 Kg/m^2^]. Most lesions were localized on the lower third of oesophagus (49%). EUS was performed on 62 patients and lymph node invasion was suspected in 62% of cases (n = 38). PET was performed in 20 patients (16%).

**Table 1:** Perioperative data

	**n**	**%**		**n**	**%**
**Total**	**120**	**100**	**Total**	**120**	**100**
Preoperative data			Histopathological findings		
Age (years)			Histology		
- ≤ 60	46	38	- Adenocarcinoma	75	62
- > 60	74	62	- Squamous cell carcinoma	45	38
Sex			pT stage		
- Male	97	81	- 0	6	5
- Female	23	19	- is	6	5
Weight loss			- 1	29	24
- ≤ 10%	96	80	- 2	21	18
- > 10%	24	20	- 3	55	46
Body Mass Index (Kg/m^2^)			- 4	3	3
- ≥ 25	74	62	pN stage		
- < 25	46	38	- 0	61	51
ASA Score			- 1, 2 or 3	59	49
- 1	51	43	Number of positive lymph nodes		
- 2	39	33	- 0	61	51
- 3	30	25	- 1-4	34	28
Risk factor			- > 4	25	21
- Gastro-oesophageal Reflux Disease	44	37	Lymph node ratio		
- Alcohol	71	59	- ≤ 0.2	88	73
- Tobacco	32	27	- > 0.2	32	27
Localisation			pM stage		
- Oesophagogastric junction	32	27	- 0	117	98
- Lower third oesophagus	59	49	- 1	3	3
- Mid third oesophagus	29	24	Differentiation		
Tumor stenosis			- undiff./poorly	23	21
- None	51	43	- moderately/well	88	79
- Incomplete	53	44	Resection		
- Complete	16	13	- R0	95	79
TDM lymph node			- R1	24	20
- No pathol. lymph node	93	77	- R2	1	1
- Pathol. lymph node	27	23	Circumferential margin		
EUS results			- Complete	99	83
usT	60		- Incomplete	21	17
- 1 & 2	33	55	Node capsular penetration		
- 3 & 4	27	45	- No	115	96
usN	60		- Yes	5	4
- 0	23	38	Angiolymphatic invasion		
- 1	38	62	- No	58	60
Preoperative treatment			- Yes	39	40
- None	83	69	Neural invasion		
- Chemotherapy	25	21	- No	54	57
- Chemo radiotherapy	12	10	- Yes	40	43
Postoperative data					
30-day morbidity	53	44			
- major	18	15			
- minor	35	29			
30-day mortality	4	3			

Neo-adjuvant CT was administered to 25 patients (21%) and CRT to 12 patients (10%). Clinical response was obtained in 76% of cases (n = 28).

### Operative results

Median length of procedures was 340 minutes [185-525 minutes]. Fifteen percent of patient required blood transfusion. Median length of oro-tracheal intubation was 8.3 hours [5.70-14.3 hours].

### Postoperative results

Median length of stay was 17 days [8-109 days]. Postoperative mortality was 3% with 15% of major complication (> 3 Clavien Dindo classification) (Table 1). Main complications were respiratory complications (15%). Anastomotic fistula rate was 6.6% (n = 8).

### Pathological findings

Adenocarcinoma was found in 62% of cases and squamous cell carcinoma in 38%. Complete resection was obtained in 79% of patients. Most R1 resections were because of positive circumferential resection margin (17%). Inferior longitudinal margin was positive in one patient (0.8%) and superior longitudinal margin in two patients (1.7%). On histopathological assessment of the resected specimens, the pT stage was: pT0 (5%), pTis (5%), pT1 (24%), pT2 (18%), pT3 (46%) and pT4 (3%). Most lesions were moderately or well differentiated (68%). Lymph node involvement was present in 49% of cases with median number of resected lymph node of 15 [4-43]. Median lymph node ratio was 0.14 [0-0.9].

### Overall survival

Four patients were lost of follow-up after a median follow-up of 47 months [28-67 months] and were kept in the study. After a median follow-up of 58 months [48.4-69.1 months], 57 patients have died. The 1- and 5-year overall survival probabilities were 86.9% (95% CI = [79.5; 91.7]) and 49.0% (95%CI = [38.3; 58.8]).

### Relapse free survival

**Figure 1:**
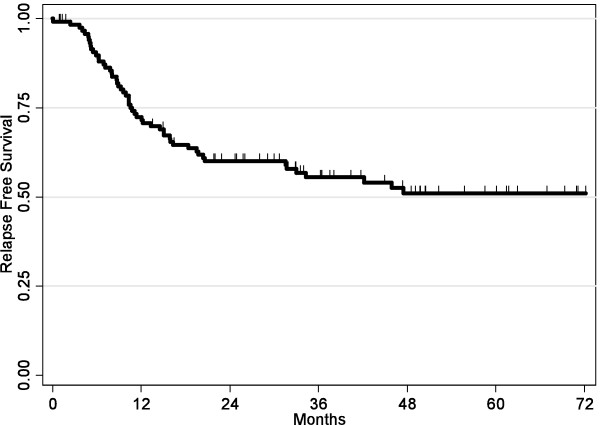
Kaplan-Meier Relapse Free Survival curve.

Fifty three patients (44%) presented recurrence during follow-up with a median delay of 10.3 months [2.4-47.5 months] without any difference according to the type of recurrence. The 1- and 5-years relapse free survival rates were respectively 73% (95% CI = [64; 80%]) and 51% (95% CI = [46; 65%]) (Figure 1). Local recurrence was the most frequent first event type (n = 30, 56%). Among them, 10 patients presented cervical recurrence. Distant and disseminated recurrence occurred respectively in 17 patients (32%) and 6 patients (11%). Most recurrences occurred during the first year (58%) and none after the fourth year of follow-up. Median survival in case of recurrence was 6.7 months [1-36 months] for local recurrence and 9.8 months [2.1: 44] for distant recurrence.

Based on univariate analysis, several risk factors for recurrence were identified (Table 2). Thus, age above 60 years (p = 0.04), loss of weight greater than 10% (p = 0.038), preoperative tumor stenosis (p < 0.0001), EUS positive lymph node (p = 0.023), oesophagogastric junction location of tumor (p = 0.005), poor tumor differentiation (p = 0.031), pT stage > 2 (p < 0.0001), positive lymph node status (p < 0.0001), incomplete resection (p = 0.002), positive circumferential margin (p = 0.0019), node capsular penetration (p = 0.021), angiolymphatic invasion (p < 0.0001), neural invasion (p < 0.0001), high number of positive lymph nodes (p < 0.0001) and lymph node ratio > 0.2 (p < 0.0001) were significantly associated with a poorer RFS.

**Table 2:** Relapse free survival - univariate analysis

**Preoperative data**	**% 5-y RFS (n) (n total = 120)**	**p**
Age (years) : <60 / >60	58.6 (16) / 45.6 (37)	0.0396
ASA score : 1-2 / 3	50.0 (41) / 55.1 (12)	0.6742
Sex : Male / Female	48.5 (46) / 61.8 (7)	0.1641
Histology : Adenocarcinoma / Squamous cell carcinoma	53.9 (34) / 43.0 (19)	0.7995
Weight loss (%) : ≤ 10 / > 10	56.3 (38) / 29.2 (15)	0.0379
Body Mass Index (Kg/m^2^) : ≤ 25 / > 25	51.2 (32) / 49.4 (21)	0.597
Gastro-oesophageal reflux disease : No / Yes	45.5 (36) / 60.4 (17)	0.2185
Tumor stenosis : No / Yes	77.9 (11) / 31.8 (42)	<0.0001
TDM pathological lymph node : No / Yes	56.0 (37) / 33.7 (16)	0.063
EUS results		
usT : 1&2 / 3&4	72.5 (9) / 52.0 (11)	0.1727
usN : 0 / 1	78.1 (4) / 51.7 (16)	0.0227
Preoperative treatment : No / CT or CRT	51.9 (37) / 49.2 (16)	0.7166
Tumor localization : Oesophagogastric jct. / Lower or mid third	40.2 (18) / 58.9 (35)	0.0046
**Postoperative data**	**% 5-y RFS (n)**	**p**
Differentiation: Undiff. or poorly diff. / Moderately or well diff.	40.9 (13) / 55.6 (35)	0.0313
pT Stage : T ≤ 2 / T > 2	75.9 (13) / 24.9 (40)	<0.0001
pN Stage : N0 / N+	82.9 (9) / 16.7 (44)	<0.0001
Resection : R0 / R1 or R2	57.2 (37) / 25.0 (16)	0.002
Circumferential margin : Complete / Incomplete	56.9 (39) / 19.6 (14)	0.0019
Node capsular penetration : No/Yes	52.4 (49) / 20.0 (4)	0.0206
Angiolymphatic invasion : No / Yes	66.4 (17) / 19.4 (29)	<0.0001
Neural invasion : No / Yes	66.5 (16) / 25.5 (28)	<0.0001
Number of positive lymph nodes : 0 / 1-4 / >4	82.9 (9) / 23.4 (23) / 7.8 (21)	<0.0001
Lymph node ratio : ≤ 0.2 / > 0.2	66.5 (26) / 0.0 (27)	<0.0001

Results of multivariate analysis are summarized on Table 3. In the multivariate analysis for pre-operative variables (Step 1), only presence of tumor stenosis was associated with an increased risk of relapse (HR = 3.36; 95% CI = [1.68; 6.72] p = 0.001).

**Table 3:** Relapse Free Survival - Multivariate analysis

	**Cox Model**			**Competing risks & Fine & Gray**							**Cox RFS**	
	**Relapse Free Survival**			**Local recurrence**			**Distant recurrence**			**Step 3 : Final analysis**		
	**HR**	**95% CI**	**p**	**HR**	**95% CI**	**p**	**HR**	**95% CI**	**p**	**HR**	**95% CI**	**p**
**Step 1 : Preoperative data**												
**Weight loss (%)**												
- ≤ 10	1			1			1					
- > 10	1.17	0.63; 2.19	0.615	0.81	0.34; 1.94	0.648	1.74	0.72; 4.22	0.218			
**Age (years)**												
- < 60	1			1			1					
- > 60	1.79	0.99; 3.23	0.051	1.13	0.55; 2.32	0.731	2.4	0.91; 6.34	0.076			
**Tumor stenosis**												
- No	1			1			1			1		
- Yes	3.36	1.68; 6.72	0.001	3.46	1.38; 8.70	0.008	2.21	0.78; 6.23	0.135	1.12	0.54; 2.39	0.757
**Step 2 : Pathological findings**												
**Tumor localization**												
- Oesophagogastric jct	1.16	0.55; 2.45	0.689	1.27	0.51; 3.16	0.61	0.92	0.30; 2.78	0.879			
- Lower/mid third	1			1			1					
**Differentiation**												
- undiff./poorly	1.03	0.47; 2.28	0.933	0.32	0.07; 1.33	0.116	3.22	1.03; 10.04	0.044			
- moderately/well	1			1			1					
**T Stage**												
- pT ≤ 2	1			1			1			1		
- pT > 2	2.91	1.16; 7.31	0.023	1.03	0.28; 3.82	0.955	5.86	0.93; 37.15	0.06	2.42	1.22; 4.79	0.011
**N Stage**												
- pN0	1			1			1			1		
- pN+	4.67	1.47; 14.77	0.009	6.01	0.84; 43.10	0.074	3.48	0.68; 17.81	0.135	3.69	1.53; 8.96	0.004
**Resection**												
- R0	1			1			1					
- R1 or R2	0.52	0.24; 1.13	0.098	0.99	0.35; 2.74	0.98	0.4	0.11; 1.48	0.174			
**Lymph node ratio**												
- ≤ 0.2	1			1			1			1		
- > 0.2	2.71	1.34; 5.51	0.006	1.25	0.46; 3.44	0.659	2.45	0.81; 7.38	0.112	2.57	1.38; 4.76	0.003
**Angiolymph. invasion**												
- No	1			1			1					
- Yes	1.98	0.96; 4.09	0.066	1.7	0.58; 5.01	0.329	0.95	0.33; 2.75	0.927			
**Neural invasion**												
- No	1			1			1					
- Yes	1.14	0.55; 2.38	0.729	1.3	0.43; 3.91	0.64	0.77	0.22; 2.67	0.684			

For pathological data (Step 2), the following variables were associated with a higher probability of relapse in multivariate analysis: pT stage > 2 (HR = 2.91; 95% CI = [1.16; 7.31] p = 0.023), positive lymph node status (HR = 4.67; 95% CI = [1.47; 14.77] p = 0.009) and lymph node ratio > 0.2 (HR = 2.71; 95% CI = [1.34; 5.51] p = 0.006).

**Figure 2:**
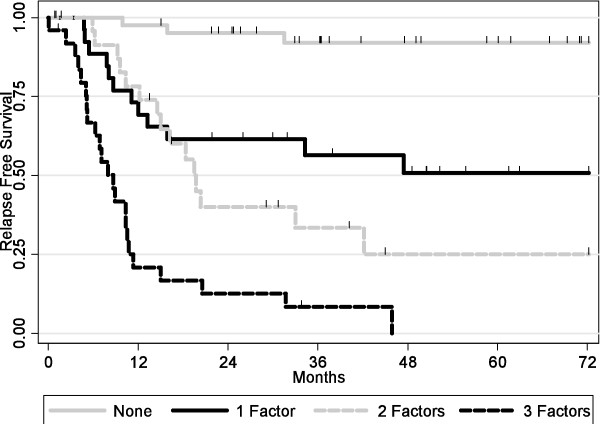
Kaplan-Meier Relapse Free Survival curves according to the number of risk factors (p < 0.001).

When combining pre-operative and pathological variables statistically significative in the two previous multivariate analysis (Step 3), the following variables were associated with an increased risk of relapse: pT stage > 2 (HR = 2.42; 95% CI = [1.22; 4.79] p = 0.011), positive lymph node status (HR = 3.69; 95% CI = [1.53; 8.96] p = 0.004) and lymph node ratio > 0.2 (HR = 2.57; 95% CI = [1.38; 4.76] p = 0.003). Relapse free survivals according to the number of risk factors were presented on Figure 2 (p < 0.0001). Addition of risks factors was correlated with a poorer RFS.

### Competing risks analysis

In multivariate analysis (Table 3), only the presence of preoperative tumor stenosis was associated with an increased risk of local recurrence (HR = 3.46; 95% CI = [1.38; 8.70] p = 0.008). There was a trend for presence of positive lymph node (HR = 6.01; 95% CI = [0.84; 43.10] p = 0.074) that did not reach statistical significance. Concerning distant recurrence (Table 3), only the presence of poor or undifferentiated tumor was associated with a higher probability of distant recurrence (HR = 3.32; 95% CI = [1.03; 10.04] p = 0.044).

## Discussion

Several studies have identified different risk factors of tumor recurrence after surgery of oesophageal cancer. Positive lymph node status is the most frequent factor identified [7-9]. It is also highlighted as an independent risk factor in our study. However, the impact of lymph node involvement can be refined by studying the prognostic role of lymph node ratio ie ratio between positive lymph nodes and resected lymph nodes. Ratios varying from 0.1 to 0.5 have already been discussed [8,10]. In this study, ratio was set at 0.2, low enough to be discriminating. It turns out to be an independent major risk factor for relapse free survival that can be illustrated by a 5-year RFS of 67% with a ratio ≤ 0.2 versus 0% with a ratio > 0.2. An interesting finding of our study is the independency of lymph node ratio as a risk factor, particularly independent from lymph node positivity what could have been a confusing factor.

Other authors have also identified the number of involved nodes as a prognostic factor [9,11]. These studies show the importance of node involvement as a quantitative and not only qualitative factor and are behind the recent changes to the 2009 UICC TNM classification. This last distinguishes nowadays different stages of nodal involvement for oesophageal cancer, superimposed on the oesophagogastric junction cancer classification, whereas it included only a single stage of nodal involvement in the previous version [12].

Unfortunately, information on the presence of this major prognostic factor is only obtained retrospectively on specimen examination. It does not allow optimal adaptation of neo-adjuvant treatment. Current diagnostic methods combining computed tomography, EUS and endoscopy permit an appropriate staging in 56% of cases and adjunction of PET does not provide better sensitivity for the preoperative lymph node diagnosis compared to EUS or computed tomography (sensitivity of 0.57 vs 0.8 and 0.5 resp.) [13,14].

Indication of adjuvant treatment after oesophageal surgery is not recommended even in case of lymph node involvement but may be discussed if the patient is healthy, demanding, and informed. However, lymph node invasion seems to be a very accurate predictor of relapse free survival and may be used to identify patients requiring adjuvant chemotherapy or chemoradiotherapy to treat systemic metastases developing after primary resection [15].

Tumor depth, defined with pT stage, was also found as an independent prognostic factor in our study. Depth invasion has also been described in several other studies as a prognostic factor independent of nodal status [16,17]. Similarly, the importance of tumor length or its development location have been described [18].

Preoperative tumor stenosis was found in our study to be an independent risk factor of local relapse that, to our knowledge, has never been previously reported. This is the main new factor identified in our study. The interest of this factor, simply defined on endoscopic and/or barium swallow criteria, is its preoperative availability. Despite its clear clinical correlation with a high pT stage, quality of resection but also with nutritional status, it appears, statistically, to be an independent risk for local relapse and not for distant relapse. It could represent an additional argument in the discussion of either preoperative chemoradiotherapy or in the indication of transthoracic vs transhiatal approach to get an optimal local disease control. Indeed, there is evidence that preoperative chemoradiotherapy increases the rate of complete resection i.e. local control for patients with locally advanced disease even if this was not always translated into a survival benefit in individual studies [19,20]. Similarly, transthoracic approach allows greater lymphadenectomy (eight more lymph node retrieved compared with transhiatal approach) [21] that might provide increased local control and disease free survival [22].

Several other risk factors of recurrence, very different, have been described as circumferential margin involvement [23,24], involvement of subcarinal and lower paraoesophageal lymph nodes [25], tumor-stroma ratio [26], endoscopic tumor length [27], good performance status of the patient [28]. Even if tumor stage and lymph node ratio are probably the most relevant risk factors of recurrence, this kind of studies, as ours, are necessary to refine oesophagus cancer prognosis and to help in the decision for adjuvant therapy.

Locoregional recurrence has been described with longer survival than distant or disseminated recurrence particularly in case of cervical recurrence [29,30]. On the other hand, Bhansali *et al*. [31] reported no difference in different types of recurrence. In our report, survival in case of local recurrence was surprisingly 3 months shorter than for distant recurrence. This is probably due to differences in treatment protocol after relapse.

Half of our patients presented with pT < 3 or negative preoperative lymph node status. That partly explains our low rate of patient who received preoperative treatment (30%). Moreover, the length of our study, begun in 1998, is another explanation, first main studies of neo-adjuvant treatment still not published at that time. Finally, low rate of 50% of preoperative EUS performed may have led to a preoperative downstaging and also explains the rate of preoperative treatment.

A same approach was performed with overall survival and brought similar results. As our study focused on recurrence risk, these results are not detailed here. Five year overall survival estimated by Kaplan Meier method was 49% in our survey. This result, higher than OS reported in literature [1], may be due to our high rate of low stage tumors and drastic patient selection for surgical treatment.

Adenocarcinoma and squamous cell carcinoma were both included in this study that may be a bias. Indeed, Siewert *et al.*[32] reported two different diseases with different pathogenesis, epidemiology, tumor biology and prognosis requiring different therapeutic strategies. However, focusing on relapse and survival, several studies comparing adenocarcinoma and squamous cell carcinoma have reported worse, similar or better survival according to histopathology [33-35]. In our study, histopathology doesn’t appear to be correlated to RFS and OS and multivariate analysis allowed us to statistically study all of our patients in the same cohort. Signet ring cell adenocarcinoma is well known to have poorer prognosis [36]. Our number of patient and particularly with signet ring cell was too small to study it as a risk factor.

This study might initiate thinking for patients who received neoadjuvant treatment as indicated in guidelines, if they could benefit, in presence of these risks factors, of post-operative treatment or of a more intensive clinical or radiological follow-up. Moreover, recurrence risk is directly correlated with the number of positive risk factors (pN stage, pT stage and lymph node ratio), creating a risk score of recurrence, what may ponder discussion according to this score. Our score has to be confirmed by others studies but strongly argue for adjuvant therapy in case of cumulated risks, particularly in case of tumor regression after preoperative treatment.

Limitations of this study are numerous. The main is it retrospective type, while conducted on a homogeneous group of patients, that has led to heterogeneity in preoperative administrated treatments. These results have to be confirmed in a prospective one with standardized protocols. Then, monocentric aspect of the study allowed us to analyze patients with standardized practices but represents also a bias as the results must be confirmed in multicentric studies. Besides, our department is not a high volume center for oesophageal surgery, due to a low incidence of oesophageal cancer in our county, but belongs to the university hospital and is experienced in oesophago-gastric surgery.

## Conclusion

Finally, our study demonstrates that preoperative tumor stenosis is the only prognostic factor associated with an increased risk of local relapse. Concerning relapse free survival, pT stage > 2, positive lymph node status and lymph node ratio > 0.2 are independent prognostic factors of recurrence after Ivor-Lewis surgery for cancer. Furthermore, addition of these risk factors is correlated with an increasing risk of recurrence. Thus, a routine postoperative treatment or reinforced follow-up should probably be considered in patients combining these risk factors.

### Consents

Written informed consent was obtained from the patients included or their family for the publication of this study.

## Abbreviations

ASA: American society of anesthesiologists; BMI: Body mass index; CT: Chemotherapy; CRT: Chemoradiation therapy; EUS: Ultrasonographic endoscopy; OS: Overall survival; PET: Positron emission tomography; RFS: Relapse free survival.

## Competing interests

The authors declare that they have no competing interests.

## Authors’ contributions

MCR was responsible for the acquisition, analysis and interpretation of data, and wrote the manuscript. NC supervised the study, and assisted in writing the manuscript. TF was responsible for the statistical analysis. BB, MH, CHJ, EB, LG, SK, GP and BP assisted in data acquisition. All authors read and approved the final manuscript.
